# Is the whole larger than the sum of its parts? Impact of missing data imputation in economic evaluation conducted alongside randomized controlled trials

**DOI:** 10.1007/s10198-020-01166-z

**Published:** 2020-02-27

**Authors:** Bernhard Michalowsky, Wolfgang Hoffmann, Kevin Kennedy, Feng Xie

**Affiliations:** 1 German Center for Neurodegenerative Diseases (DZNE), Site Rostock/Greifswald, Ellernholzstrasse 1-2, 17487 Greifswald, Germany; 2 Department of Health Research Methods, Evidence and Impact (Formerly Clinical Epidemiology and Biostatistics), McMaster University, 1280 Main Street West, Hamilton, Canada; 3 Program for Health Economics and Outcome Measures (PHENOM), Hamilton, Canada; 4 Centre for Health Economics and Policy Analysis, McMaster University, 1280 Main Street West, Hamilton, Canada; 5 Institute for Community Medicine, Section Epidemiology of Health Care and Community Health, University Medicine Greifswald (UMG), Ellernholzstrasse 1-2, 17487 Greifswald, Germany

**Keywords:** Missing data, Multiple imputation, Cost-effectiveness analysis, Cost–utility analysis, C18, C43, I1, I10

## Abstract

Outcomes in economic evaluations, such as health utilities and costs, are products of multiple variables, often requiring complete item responses to questionnaires. Therefore, missing data are very common in cost-effectiveness analyses. Multiple imputations (MI) are predominately recommended and could be made either for individual items or at the aggregate level. We, therefore, aimed to assess the precision of both MI approaches (the item imputation vs. aggregate imputation) on the cost-effectiveness results. The original data set came from a cluster-randomized, controlled trial and was used to describe the missing data pattern and compare the differences in the cost-effectiveness results between the two imputation approaches. A simulation study with different missing data scenarios generated based on a complete data set was used to assess the precision of both imputation approaches. For health utility and cost, patients more often had a partial (9% vs. 23%, respectively) rather than complete missing (4% vs. 0%). The imputation approaches differed in the cost-effectiveness results (the item imputation: − 61,079€/QALY vs. the aggregate imputation: 15,399€/QALY). Within the simulation study mean relative bias (< 5% vs. < 10%) and range of bias (< 38% vs. < 83%) to the true incremental cost and incremental QALYs were lower for the item imputation compared to the aggregate imputation. Even when 40% of data were missing, relative bias to true cost-effectiveness curves was less than 16% using the item imputation, but up to 39% for the aggregate imputation. Thus, the imputation strategies could have a significant impact on the cost-effectiveness conclusions when more than 20% of data are missing. The item imputation approach has better precision than the imputation at the aggregate level.

**Electronic supplementary material:**

The online version of this article (10.1007/s10198-020-01166-z) contains supplementary material, which is available to authorized users.

## Introduction

Cost–utility analyses (CUA) conducted alongside randomized controlled trials are commonly used approaches to generate cost-effectiveness evidence [1]. Utility-based instruments and resource use questionnaires administered alongside these trials usually consist of multiple questions to which complete responses are needed to calculate health utility and the total cost. However, even carefully designed and well-executed trials contain missing responses from individual participants [2]. Therefore, missing data are common and, depending on the proportion and nature of the missing (completely at random, at random, and not at random), could affect the precision and accuracy of cost-effectiveness results [2–4].

About 43% of the economic evaluations have restricted the analysis to those patients with complete data [5]. The exclusion of individuals with missing values could bias the cost-effectiveness conclusion, especially if the data are missing at random [6, 7]. Therefore, simple methods, such as mean or median imputation, or multiple imputations (MI) are used to handle missing data. Both methods worked well to handle cost data that are missing completely at random, but MI performed better when the data are missing at random [5, 7]. Thus, MI is usually recommended [2–4, 8–10] and, therefore, has been used in one-third of economic evaluations [5].

Whereas utility-based questionnaires usually consist of five or six questions [11, 12], resource utilization questionnaires could consist out of 20 and more questions about used healthcare services. Therefore, it is more likely to see missing responses in these questionnaires. These missing responses can be imputed individually and then used to calculate health utility or total cost (referred to as “*item imputation*”). Alternatively, only the health utility and the total cost can be imputed, whenever there is any missing value (referred to as “*aggregate imputation*”).

Simons et al. [13] revealed that in large samples (*n* > 500) and a missing data pattern that follows mainly a unit non-response (referring to the complete absence of an interview/ assessment), the item and aggregate imputation of missing data of the EQ-5D produced similar results. However, item imputation became more accurate with a pattern of missingness following an item non-response (referring to the absence of some answers to specific questions in the interview/assessment) and in smaller samples (*n* < 100). Eekhout et al. [14] evaluated the performance of both imputation methods for handling missing data for a 12-item instrument and found that when a large percentage of subjects had missing items (> 25%), the item imputation outperformed the aggregate imputation. Thus, for costs, there may be an advantage to impute on the individual resource use item level, especially when there are only a few cost drivers.

In economic evaluations, costs and QALYs were jointly used to estimate the incremental cost-effectiveness ratio (ICER). Therefore, our primary objective was to assess the impact of the item and aggregate imputation methods using MI on ICER and resulting cost-effectiveness acceptability curve (CEAC).

## Materials and methods

### Overview

The original data came from a cluster-randomized, controlled intervention trial of 407 patients followed up over a 12-month time frame. We first demonstrated the missing data pattern at the item and the aggregate level and compared the differences in cost-effectiveness results between the imputation approaches. Then we used a subset of 289 patients who did not have any item missing (i.e., complete cases) to simulate different missing data scenarios reflecting different magnitudes (10%, 20%, and 40% of the aggregated outcomes, i.e., cost and QALYs) and patterns (completely at random, at random, and not at random) of missing. Each scenario was replicated 300 times to increase the robustness of results. Within each replication, we used MI by Chained Equations (MICE) to impute (a) missing responses to SF-6D and resource use questions individually and (b) health utility and total cost at the aggregated level [6, 15, 16]. Subsequently, for each replicated scenario incremental cost, incremental QALYs, ICER, and CEAC were calculated. Finally, the deviation (relative bias) from true incremental cost, incremental QALYs, and the probability of cost-effectiveness at a wide range of willingness-to-pay (WTP) thresholds (0€ to 250,000€) were calculated [17, 18]. Results were displayed using scatter plots with density rugs and CEACs.

### Trial design, setting and sample

The original DelpHi trial (Dementia: life- and person-centered help) was a general practitioner (GP)-based, cluster-randomized controlled intervention trial in a primary care setting in Germany. The study design [19, 20], sample [21], primary outcome [22], and the economic evaluation [23] have been published elsewhere. The DelpHi trial was approved by the Ethical Committee of the Chamber of Physicians of Mecklenburg-Western Pomerania, registry number BB 20/11.

Overall, 634 participants agreed to participate, 516 participants started the baseline assessment, and 407 completed the first follow-up assessment. Totally, 118 (29%) patients had at least one missing at baseline or follow-up. Therefore, the simulation study was based on a complete data set of 289 patients. A detailed description of the study characteristic is presented in Supplementary Table 1.

### Healthcare resource utilization, costs and health utilities

A standardized computer-assisted interview was conducted to collect data on patients’ healthcare resource utilization retrospectively for 12 months using proxy ratings. The resource utilization questionnaire consists of 23 items, including medical treatments and care services. Mean costs per patient were calculated using published unit costs in 2018 Euros (€) [24, 25]. Assumptions for the calculation of costs are reported in Supplementary Table 2.

Health-related quality of life (HRQoL) was assessed using the 12-Item Short-Form Health Survey (SF-12), a generic, multidimensional instrument. SF-12 measures the physical and mental dimensions of HRQoL [26]. Eight responses of the SF-12 were converted to health utilities, a single index measure for HRQoL anchored at 0 for death and 1 for full health [26, 27]. By assuming a linear change of HRQoL, we used the health utilities at baseline and the 12-month follow-up to calculate the QALY for each patient using the area under the curve approach. A description of health utilities, QALYs as well as incremental cost, incremental QALYs, and ICER of the complete data set is demonstrated in Supplementary Table 3.

### Multiple imputation methods

We used MICE to impute (a) each missing individual response or (b) the health utility and the total cost [6, 15, 16]. Through MICE, Poisson regression was used for each missing resource utilization variable, an ordered logistic regression for each SF-6D response, and linear regression models for health utility and total cost, respectively. To account for the stochastic dependency of patients treated by the same GP, GPs were included as random effects. Each model was adjusted for age, sex, living situation (alone or not alone), comorbidity (number of ICD-10 diagnoses) and functional impairment according to the Bayer Activities of Daily Living Scale (B-ADL) [28]. 50 values were estimated by MICE for each missing value. Estimates obtained from each imputed value were combined using Rubin’s rule [29] to generate a mean estimate and standard error [30]. Furthermore, MICE was implemented separately by treatment group [6]. A description of the imputation process and the used STATA code are presented in Supplementary Document 1.

### Missing patterns in the original DelpHi trial data

We demonstrated the missing data patterns on item and aggregate level for the original dataset of 407 patients in Table 1. A missing was more likely in patients with higher functional impairment (Odds Ratio 1.26, *p* = 0.001) as shown in the missing data analysis (see Supplementary Table 4). We calculated the incremental cost, incremental QALYs, and ICER of the complete cases (*n* = 289), as well as results with the imputation at the item and aggregate levels.

**Table 1:** Description of missing resource utilization and SF-6D data

	Overall	Intervention	Control	
	*n* = 407	*n* = 291	*n* = 116	*p *value
**SF-6D and resource utilization questionnaires*, n (%)***				
Patients who completely respond	289 (71.0%)	199 (68.4%)	90 (77.6%)	0.070
Patients who had a complete missing for all items	0 (0.0%)^a^	0 (0.0%)^a^	0 (0.0%)^a^	1.000
Patients who had a missing at least in one item	118 (29.0%)	92 (31.6%)	26 (22.4%)	0.070
**SF-6D questionnaire*, n (%)***				
Patients who completely respond	352 (86.5%)	251 (86.2%)	101 (87.1%)	0.874
Patients who had a complete missing for all items	18 (4.4%)	14 (4.8%)	4 (3.5%)	0.790
Patients who had a missing at least in one item	37 (9.1%)	40 (13.8%)	15 (12.9%)	0.874
Missing item physical functioning	23 (5.7%)	17 (5.8%)	6 (5.2%)	1.000
Missing item role participation	29 (7.1%)	19 (6.5%)	10 (8.6%)	0.522
Missing item social functioning	28 (6.9%)	19 (6.5%)	9 (7.7%)	0.667
Missing item bodily pain	39 (8.0%)	28 (9.62%)	11 (9.5%)	1.000
Missing item mental health	26 (6.4%)	19 (6.5%)	7 (6.0%)	1.000
Missing item vitality	25 (6.1%)	21 (7.2%)	4 (3.4%)	0.177
**Resource utilization questionnaire, *n (%)***				
Patients who completely respond, *n* (%)	315 (77.4%)	221 (76.0%)	94 (81.0%)	0.295
Patients who had a complete missing for all items	0 (0.0%)^a^	0 (0.0%)^a^	0 (0.0%)^a^	1.000
Patients who had a missing at least in one item	92 (22.6%)	70 (24.0%)	22 (19.0%)	0.295
Missing item ambulatory care	29 (7.1%)	3 (1.0%)	0 (0.0%)	0.561
Missing item day and night care	31 (7.6%)	28 (9.6%)	3 (2.6%)	0.013
Missing item hospital treatments	40 (9.8%)	32 (11.0%)	8 (6.9%)	0.268
Missing item rehabilitation	28 (6.9%)	24 (8.2%)	4 (3.4%))	0.126
Missing item cure	30 (7.4%)	25 (8.6%)	5 (4.3%)	0.205
Missing item medication/drugs	5 (1.2%)	2 (0.7%)	3 (2.6%)	0.142
Missing item medical aids	13 (3.2%)	12 (4.1%)	1 (0.9%)	0.121
Missing item therapies	46 (11.3%)	36 (12.4%)	10 (8.6%)	0.305
Missing item nursing care	0 (0.0%)	0 (0.0%)	0 (0.0%)	1.000
Missing item general practitioner	53 (13.0%)	44 (15.1%)	9 (7.8%)	**0.050**
Missing item internist	57 (14.0%)	47 (16.2%)	10 (8.6%)	0.057
Missing item neurologist	56 (13.7%)	46 (15.8%)	10 (8.6%)	0.078
Missing item gynecologist	42 (10.3%)	34 (11.7%)	8 (6.9%)	0.479
Missing item surgeon	57 (14.0%)	47 (16.2%)	10 (8.6%)	0.057
Missing item orthopaedist	58 (14.3%)	48 (16.5%)	10 (8.6%)	**0.041**
Missing item urologists	56 (13.8%)	46 (15.8%)	10 (8.6%)	0.078
Missing item ear, nose and throat specialist	55 (13.5%)	45 (15.5%)	10 (8.6%)	0.077
Missing item ophthalmologist	56 (13.8%)	45 (15.5%)	10 (8.6%)	0.077
Missing item dermatologist	54 (13.3%)	45 (15.5%)	9 (7.8%)	0.059
Missing item psychiatrist	55 (13.5%)	45 (15.5%)	10(8.6%)	0.077
Missing item dentist	52 (12.8%)	43 (14.8%)	9 (7.8%)	0.069
Missing item other specialists 1	58 (14.3%)	48 (16.5%)	10 (8.6%)	**0.041**
Missing item other specialists 2	57 (14.0%)	47 (16.2%)	10 (8.6%)	0.057

^a^Utilization of institutionalization (nursing home care) was assessed by patients living situation (could be assessed without the patient or the caregiver) and was, therefore, not included in this ratio

*p* values less than 0.05 are highlighted in bold

### Simulation study: constructing the missing data scenarios

Using the complete dataset of 289 patients without any item missing, we randomly constructed different missing data scenarios to reflect different magnitudes (10%, 20%, and 40%) and patterns of missing data. Specifically, 1.25%, 2.5% and 5% of SF-6D responses and resource utilization item were randomly removed (according to a specific missing data pattern as described below), resulting in an average missing of 10% (range 7–17%), 20% (range 13–26%) and 40% (range 32–47%) at the aggregate level (i.e., health utility and total cost). Generated missing data scenarios resulted in missing data patterns with only a few items per patient missing, not in a complete missing. A detailed description of the randomly generated missing data patterns is represented in Supplementary Table 5.

For missing completely at random, values were randomly deleted. For the missing at random data pattern, missing data were more likely in patients having higher comorbidity (number of listed ICD-10 diagnoses) and higher deficits in daily living activities according to B-ADL [28]. For missing not at random, patients with a high resource utilization were more likely to have missing values. Thus, in this scenario, missing values were more common in high-cost patients. Overall, nine missing data scenarios were created (i.e., three missing patterns x three proportions of missing data). To avoid the results being influenced by one particular data set, for each of the nine imputation scenarios, we randomly generated 300 datasets.

### Simulation study: cost-effectiveness and statistical analysis

For each replication, the incremental cost, incremental QALY, and ICER were calculated [31–33]. To handle sampling uncertainty in the ICER, we used nonparametric bootstrapping [34]. The probability of the DCM being cost-effective was calculated using a wide range of WTP thresholds (0€ to 250,000€) [17, 18].

The following outcomes were used to assess the accuracy and precision of the item imputation and the aggregated imputation by comparing those with the true values of the complete data set of 289 patients without any item missing.

i. Relative bias: The deviation from true incremental cost and incremental QALY in percent was calculated by averaging the 300 replications of each scenario. The relative bias (for example, for incremental cost) was calculated in percent as follows:
  $${\mathrm{R}\mathrm{e}\mathrm{l}\mathrm{a}\mathrm{t}\mathrm{i}\mathrm{v}\mathrm{e} \,\,\mathrm{b}\mathrm{i}\mathrm{a}\mathrm{s}}_{\mathrm{I}\mathrm{C}}=\frac{\frac{1}{300}{\sum }_{i=0}^{300}{\Delta C}_{i}}{{\Delta \mathrm{C}}_{\mathrm{t}\mathrm{r}\mathrm{u}\mathrm{e}}}-1,$$
  $${\Delta C}_{i}\,\, \mathrm{i}\mathrm{s}\,\, \mathrm{t}\mathrm{h}\mathrm{e}\,\, \mathrm{i}\mathrm{n}\mathrm{c}\mathrm{r}\mathrm{e}\mathrm{m}\mathrm{e}\mathrm{n}\mathrm{t}\mathrm{a}\mathrm{l}\,\, \mathrm{c}\mathrm{o}\mathrm{s}\mathrm{t} \,\,\mathrm{o}\mathrm{f}\,\, \mathrm{t}\mathrm{h}\mathrm{e}\,\, \mathrm{r}\mathrm{e}\mathrm{p}\mathrm{l}\mathrm{i}\mathrm{c}\mathrm{a}\mathrm{t}\mathrm{i}\mathrm{o}\mathrm{n}\,\, i \,\,\mathrm{a}\mathrm{n}\mathrm{d}$$
  $${\Delta C}_{\mathrm{t}\mathrm{r}\mathrm{u}\mathrm{e}}\,\, \mathrm{t}\mathrm{r}\mathrm{u}\mathrm{e} \,\,\mathrm{i}\mathrm{n}\mathrm{c}\mathrm{r}\mathrm{e}\mathrm{m}\mathrm{e}\mathrm{n}\mathrm{t}\mathrm{a}\mathrm{l} \,\,\mathrm{c}\mathrm{o}\mathrm{s}\mathrm{t}.$$
ii. Range of relative bias: The 5th and 95th percentile was used to demonstrate the range of the relative bias to the true incremental cost and true incremental QALYs as well as to true CEAC.
iii. Sampling coverage probability: The sampling coverage probability represents the proportion of the 1000 non-parametric bootstrapping iterations for which the 95% confidence interval (CI) includes the true mean total cost and true QALYs. A sampling coverage probability of 1.0 indicates that true costs and QALYs are included in each of the CI of the 1000 iterations. Thus, a lower sampling coverage probability demonstrates that the imputed values are poorer estimates of the true values.
iv. Relative bias and range of bias from the true probability of cost-effectiveness: For each scenario, the mean probability at different WTP thresholds as well as the range of the probability using the 5th and 95th percentile of the replications was used to assess the deviation from the true probabilities of cost-effectiveness by averaging the 300 replications of each scenario.

Results were demonstrated descriptively and displayed using scatter plots with density rugs and CEAC. Analyses were carried out with STATA, R, and Excel.

## Results

### The missing patterns in the DelpHi trial

Four percent (*n* = 18) of the patients had a complete missing for all and 9% (*n* = 37) at least for one SF-6D item. 23% (*n* = 92) of the patients had at least one, but none patient (0%) a complete missing in all 23 resource utilization items. A description of the missing data pattern of the original data set on both levels is presented in Table 1.

Overall, using both MI approaches resulted in higher cost (10,547€ and 10,402€ vs. 7,942€) and lower QALYs (0.709 and 0.725 vs. 0.771) as compared to the complete case analysis. This was due to the fact that patients with higher functional impairment more likely had missing data in this study. These patients usually have higher treatment and care needs and thus, higher healthcare costs and lower QALYs as compared to patients without any physical limitations. Furthermore, whereas both MI approaches resulted in similar cost estimates for the intervention group (10,547€ vs. 10,402€), there were substantial differences in the cost estimates for the control group (11,348€ vs. 8,196€), leading to the differences in the ICER. The cost of the control group was much higher due to the fact that much of the available information about healthcare resources used was not used. Cost for ambulatory care services (*n* = 16, mean costs 7,993€), day and night care services (*n* = 7, mean costs 5,546 €) or nursing home care (*n* = 7, mean costs 7,315€) in moderately to severely functionally impaired patients was not taken into account in the complete case analysis. However, these resources represent the effect of the intervention, which was intended to delay the progression of dementia diseases and, thus, the utilization of healthcare services. However, it seems that this interventional effect could explain the higher costs in the controls, but QALYs did not differ significantly between both groups after imputing missing items or the aggregated outcomes. Therefore, ICER of the complete case, the aggregated and the item imputation valued 129,002€/QALY, 15,399€/QALY and − 61,079€/QALY, respectively (see Table 2).

**Table 2:** Incremental cost, incremental QALY, and ICER of using the complete dataset and multiple imputations at the item and aggregate level

	Cost		QALYs		∆Cost	∆QALY	ICER
	Intervention	Control	Intervention	Control			
Complete dataset (*n *= 289)	7,942€	6,632€	0.771	0.761	1,311€	0.010	129,002€/QALY
Item imputation (*n* = 407)	10,547€	11,348€	0.709	0.722	− 801€	0.013	− 61,079€/QALY
Aggregate imputation (*n* = 407)	10,402€	8,196€	0.725	0.711	2,205€	0.014	15,399€/QALY

*QALYs* quality-adjusted life years, ***∆****Cost* incremental costs, ***∆****QALY* incremental QALYs, *ICER* incremental cost-effectiveness ratio

### Simulation study

**Fig. 1:**
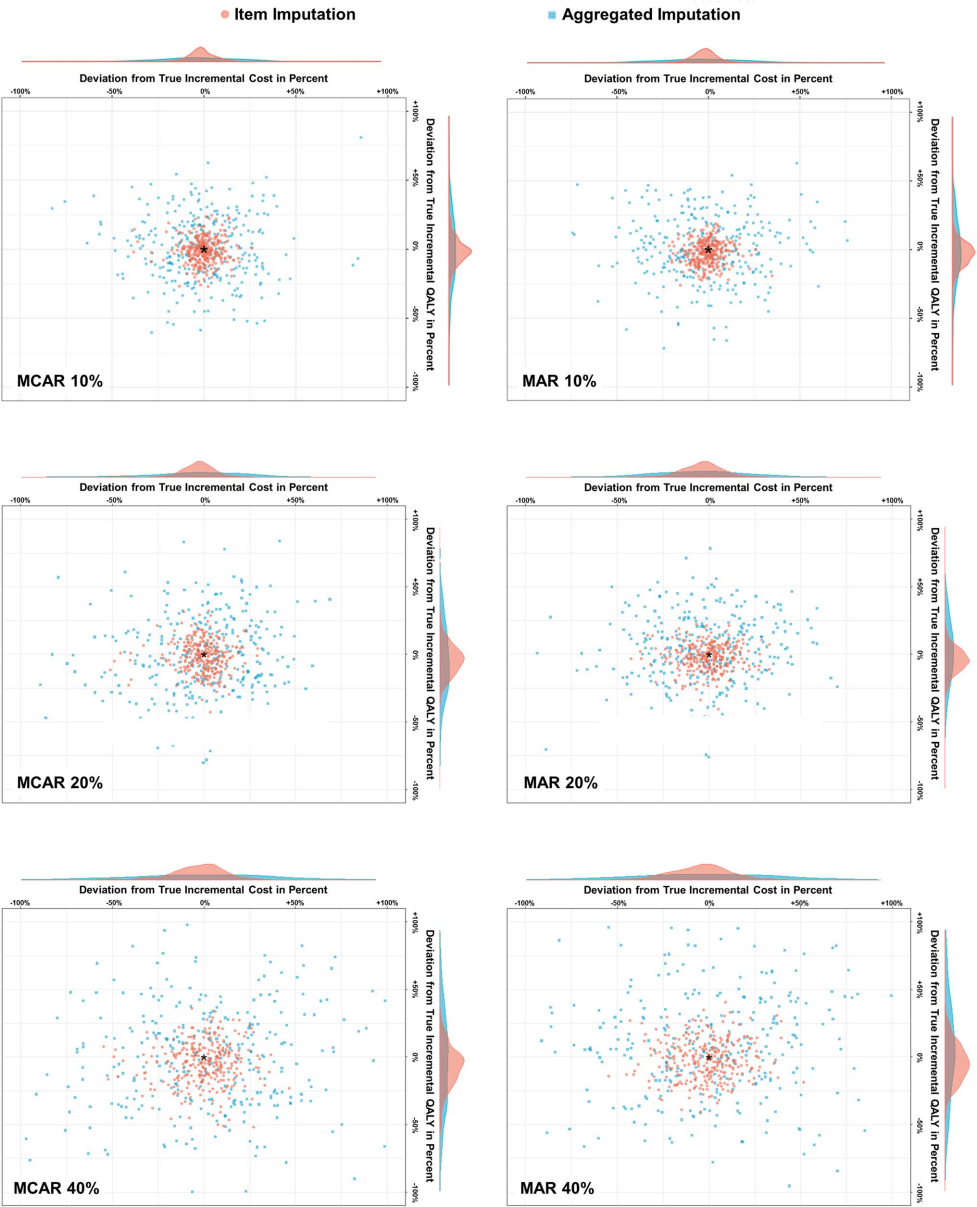
Deviation of estimated values using the item and aggregate imputations to true incremental cost and effects and density of both deviations. *MCAR* missing completely at random, *MAR* missing at random

Across all scenarios, imputing individual items was more precise and accurate than the aggregate imputation, demonstrated by a lower relative bias and a smaller range of the bias. Taking the average of all 300 replications into account, mean relative bias to the true incremental cost and incremental QALYs was lower for the item imputation, not exceeding 5% (vs. 10% for the aggregate imputation). The range of the relative bias was also wider for the aggregate imputation (up to 83%) compared to the item imputation using MI (up to 38%). Furthermore, the item imputation had a higher sampling coverage probability. When data were missing at random and 10%, 20% or 40% of data were set to be missing, the sampling coverage probabilities of the item vs. aggregate imputation were as follows: 82.8% vs. 81.7%, 82.6% vs. 81.2%, and 82.4% vs. 77.5%. The mean relative bias, the range of the bias, and the sampling coverage probabilities are shown in Fig. 1 and Table 3.

**Table 3:** Relative mean deviation in percent and range of imputed estimates to true incremental cost, effects and net monetary benefit of the item and the aggregate imputation (simulation study based on *n* = 289 patients)

	Item imputation					Aggregate imputation				
	Relative bias from true incremental cost		Relative bias from true incremental effects		Sampling coverage probability	Relative bias from true incremental cost		Relative bias from true incremental effects		Sampling coverage probability
	Mean (%)	Range^a^ (%)	Mean (%)	Range^a^ (%)	Mean	Mean (%)	Range^a^ (%)	Mean (%)	Range^a^ (%)	Mean
**Missing completely at random (MCAR)**										
10%	− 0.4	− 12−11	− 0.8	− 15−13	0.826	− 0.2	− 39−32	0.7	− 37−39	0.817
20%	− 0.7	− 18−15	− 1.6	− 21−17	0.824	− 0.6	− 52−39	− 0.7	− 47−47	0.800
40%	− 0.4	− 29−23	− 4.6	− 31−21	0.819	− 0.2	− 69−67	− 1.6	− 67–66	0.779
**Missing at random (MAR)**										
10%	− 0.6	− 13−11	− 2.1	− 13−11	0.828	− 1.2	− 41−39	0.8	− 32−35	0.818
20%	− 0.7	− 21−19	− 2.1	− 19−13	0.826	− 2.4	− 45−42	3.8	− 39−44	0.812
40%	− 1.4	− 29−25	− 1.9	− 25−24	0.824	− 1.9	− 76−67	10.1	− 56−83	0.775
**Missing not at random (MNAR)**										
10%	− 0.7	− 15−16	–	–	0.812	− 1.5	− 49−44	–	–	0.798
20%	− 0.8	− 37−25	–	–	0.782	− 1.3	− 55−55	–	–	0.740
40%	− 1.3	− 38−3	–	–	0.712	− 2.2	− 74−67	–	–	0.604

^a^The 5th and 95th percentiles were used to demonstrate the range of the relative bias to true incremental cost and QALYs

Both MI approaches were more precise when data were missing at random compared to missing completely at random, especially due to a more precise estimation of incremental QALY, demonstrated by a smaller range as compared to the incremental cost estimates. The lowest precision of the alternative imputation approaches was observed for the missing not at random scenarios, with a sampling coverage probability of up to 60.4%. However, for this pattern, the item imputation performed, again, better (81.2–71.2%) than the aggregate imputation (79.8–60.4%).

**Fig. 2:**
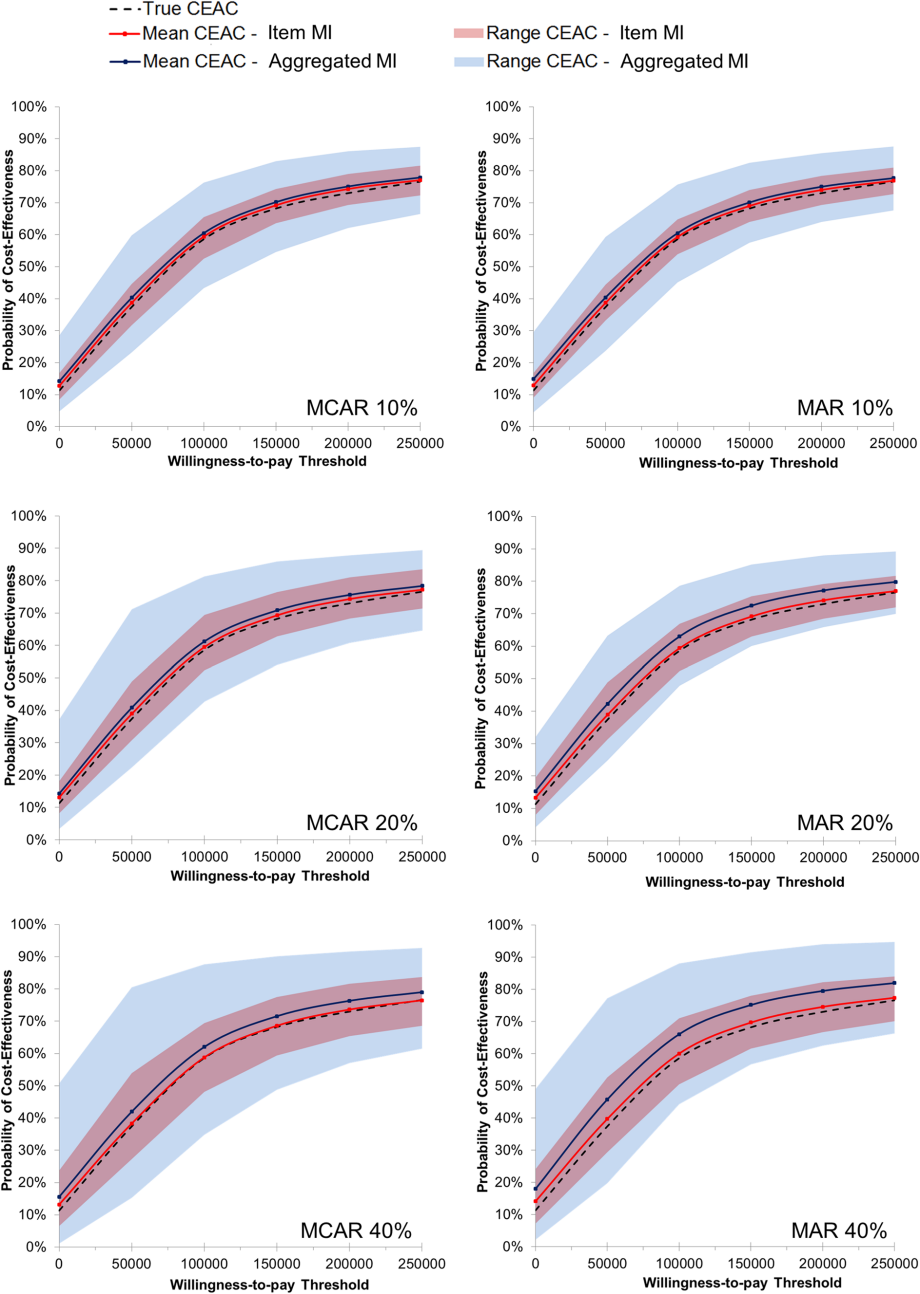
Cost-effectiveness acceptability curves of the item and aggregated imputation (simulation study based on *n* = 289 patients). *MCAR* missing completely at random, *MAR* missing at random, *CEAC* cost-effectiveness acceptability curve

The mean CEAC of the imputation at the item level was closer to the true curve (relative bias of 0–2% across all scenarios) than the CEAC of the aggregate imputation (1–8%). The range of estimated curves at different WTP thresholds was wider, especially for the aggregate imputation used in scenarios with 40% missing data. The range of bias estimated using the item imputation approach was less than 16%, even when 40% of data were missing. In contrast, the CEAC estimated using the aggregate imputation could deviate up to 39% away from the true CEAC. Relative bias and range of bias from true CEAC are demonstrated in Fig. 2 and Supplementary Table 4.

## Discussion

Most of the patients in the original DelpHi trial had a partial missing in only some items (item non-response) rather than a complete missing in all items (unit non-response), especially for the resource utilization items. Some observed information would be used if using the complete case analysis or analysis using an aggregate imputation, leading to substantial differences in the ICER. The item imputation was more likely able to capture the intervention effect, leading to a more reliable cost-effectiveness conclusion. The simulation study confirmed the advantage of the item imputation across all scenarios when the magnitude of missing is small. Even though the mean biases of estimates were low, the range of estimates could be wider, especially if the aggregate imputation was used, which might change the cost-effectiveness conclusion. The results also suggest that precision decreased with an increased amount of missing data. The lowest precision was observed for the missing not at random scenarios, where patients with a higher resource use were more likely to have missing data. The MI approaches were more precise when data were missing at random, especially when the item imputation was used.

There have been a few papers investigating how to handle missing data in cost-of-illness or cost–utility analyses. Leurent et al. [5] summarized that within the last decade more attention has been devoted to assess the reasons for missing values and to adopt methods that can incorporate missing values. MI has been proposed for handling missing data [2–4, 6, 35]. Gomez et al. [35] used fully observed data of 2078 patients and implemented different imputation methods. In this study estimated point values of the MI approach differed up to 16% from true values when there were 30% of data missing. Furthermore, Belger et al. [7] evaluated the effect of naïve and multiple imputation methods on estimated costs. The mean relative bias of the MI approach was estimated at 3% with the sampling coverage probability of 70%. These results of previously published studies are similar to the mean relative bias obtained by our aggregate imputation. However, the results of these studies represented the mean relative bias of using an aggregate imputation. As demonstrated by the range of bias in this simulation study, the aggregate imputation could substantially deviate from the true estimates, which was furthermore translated into a large deviation in CEAC.

Belger et al. [7] reported that the lowest precision of MI was revealed for missing not at random scenarios. In these scenarios, patients with higher costs were set to have more likely missing values as well, which is in line with the scenarios created in our study. The lower precision could be caused by the shape and skewness of the distribution of the cost data. Cost data are usually skewed to the right. After removing the high-cost patients, it seems to be impossible to replicate the skewed distribution of costs, resulting in an underestimation of missing cost data and thus, to a large discrepancy in the cost-effectiveness conclusion. That might also be the reason for the observed lower precision of the aggregate imputation in the missing at random scenarios in our study, where more functionally impaired and more comorbid patients were set to have missing data. In general, these patients incurred a higher cost as well. Overall, the individualized item imputation was more precise when data were missing at random. The distribution of health utilities is relatively easier to replicate compared to the cost. Therefore, further research is needed to evaluate how missing not at random data patterns as well as the estimation of highly skewed cost data could be handled in a more accurate and precise way.

Overall, this analysis highlighted the benefit of using the item imputation. The cost-effectiveness conclusion drawn after using the aggregate imputation could be different, especially when more than 20% of data were missing. Specifically to prevent a misleading decision, cost–utility studies should clearly report the proportion and the pattern of missing data at the item and aggregate levels. The item imputation may provide reasonable estimates, even when there are 40% of cases with some missing responses in the used questionnaires. In contrast, when the missingness is more likely following an item non-response rather than a complete missing (unit non-response), an aggregate imputation should not be carried out when there are more than 20% of cases missing, which could significantly mislead the cost-effectiveness conclusions. Therefore, sensitivity analyses are crucial to handle the uncertainty that is intrinsically related to the imputation methods used within economic evaluations.

### Limitations

The simulation process for creating different missing data scenarios was conducted by sampling from those individuals with complete data in the original study. There was a missing at random mechanism in the original dataset, in which patients with a higher functional impairment are more like have missing data. These missing data mechanism is explicitly considered within the simulation design, starting to generate again missing data scenarios following the initial missing at random data pattern. This initial missing data mechanism could, therefore, bias each of the simulated missing data scenarios. This limits the generalizability of the presented results. However, it is nearly impossible to obtain a complete data set, especially not in older patients. Regardless of this bias and even though the underlying missing data pattern could affect the strength of the relative bias and range of bias within both MI approaches, across all subsequently created missing data scenarios imputing individual items were consistently more precise and accurate than the alternative aggregate imputation, answering the main research question of this analysis.

Furthermore, the number of participants in the complete dataset was moderate, but unequally distributed between the intervention and the control group, especially due to a higher drop out of moderately and severely functionally impaired patients in the control group. Therefore, the controls were less likely functionally impaired and had less likely missing values, especially for the resource utilization values. Even though the MI procedure was implemented separately by randomization treatment allocation, estimates of the control group due to the lower number of patients or the intervention group due to the higher number of initially missing values could more likely be biased, leading naturally to deviating incremental cost and QALY.

Also, missing data scenarios were generated randomly but still in accordance with the determined missing data mechanism. Therefore, missing data could occur for only one or for several HRQoL or cost items. The average number of items missing per case could affect the estimated incremental cost-effectiveness ratios, and thus the conclusion about the performance of both MI approaches. The missing data scenarios of this simulation study resulted in missing data patterns that have only a few items per case were missing, not a complete missing of all items. In cases were all items of the questionnaires were missing, both approached would perform equally. Therefore, presented results are only generalizable for data with some items missing, rather than for data sets were mainly complete cases were missing. For cases where all items are missing, both MI approaches would perform comparably. Further research is needed to evaluate how many items have to be missing that the individualized imputation is beneficial compared to the aggregated imputation approach or to be more precisely, for how many items missing both MI approaches perform equally.

In addition, we used different multiple regression models and assumptions for each variable, which could furthermore influence the differences in incremental estimates in both MI approaches.

## Electronic supplementary material

Below is the link to the electronic supplementary material.

Supplementary file1 (DOCX 80 kb)

## References

[1] George B, Harris A, Mitchell A (2001). Cost-effectiveness analysis and the consistency of decision making: evidence from pharmaceutical reimbursement in Australia (1991 to 1996). Pharmacoeconomics.

[2] Briggs A (2003). Missing… presumed at random: cost-analysis of incomplete data. Health Econ..

[3] Blough DK (2009). The impact of using different imputation methods for missing quality of life scores on the estimation of the cost-effectiveness of lung-volume-reduction surgery. Health Econ..

[4] Oostenbrink JB, Al MJ (2005). The analysis of incomplete cost data due to dropout. Health Econ..

[5] Leurent B, Gomes M, Carpenter JR (2018). Missing data in trial-based cost-effectiveness analysis: an incomplete journey. Health Econ..

[6] Faria R (2014). A guide to handling missing data in cost-effectiveness analysis conducted within randomised controlled trials. Pharmacoeconomics.

[7] Belger M (2016). How to deal with missing longitudinal data in cost of illness analysis in Alzheimer's disease-suggestions from the GERAS observational study. BMC Med. Res. Methodol..

[8] Noble SM, Hollingworth W, Tilling K (2012). Missing data in trial-based cost-effectiveness analysis: the current state of play. Health Econ..

[9] White IR, Thompson SG (2005). Adjusting for partially missing baseline measurements in randomized trials. Stat. Med..

[10] Seaman SR, Bartlett JW, White IR (2012). Multiple imputation of missing covariates with non-linear effects and interactions: an evaluation of statistical methods. BMC Med. Res. Methodol..

[11] Hurst NP (1997). Measuring health-related quality of life in rheumatoid arthritis: validity, responsiveness and reliability of EuroQol (EQ-5D). Br J. Rheumatol..

[12] Johnson JA, Coons SJ (1998). Comparison of the EQ-5D and SF-12 in an adult US sample. Qual. Life Res..

[13] Simons CL (2015). Multiple imputation to deal with missing EQ-5D-3L data: should we impute individual domains or the actual index?. Qual. Life Res.

[14] Eekhout I (2014). Missing data in a multi-item instrument were best handled by multiple imputation at the item score level. J. Clin. Epidemiol..

[15] van Buuren S (2007). Multiple imputation of discrete and continuous data by fully conditional specification. Stat. Methods Med. Res..

[16] Schafer JL, Graham JW (2002). Missing data: our view of the state of the art. Psychol. Methods.

[17] Neumann PJ, Cohen JT, Weinstein MC (2014). Updating cost-effectiveness–the curious resilience of the $50,000-per-QALY threshold. N Engl. J. Med..

[18] Grosse SD (2008). Assessing cost-effectiveness in healthcare: history of the $50,000 per QALY threshold. Expert Rev. Pharmacoecon. Outcomes Res..

[19] Thyrian JR (2012). Life- and person-centred help in Mecklenburg-Western Pomerania, Germany (DelpHi): study protocol for a randomised controlled trial. Trials.

[20] Thyrian JR (2016). Community-dwelling people screened positive for dementia in primary care: a comprehensive, multivariate descriptive analysis using data from the DelpHi-study. J. Alzheimers Dis..

[21] Michalowsky B (2018). Healthcare utilization and costs in primary care patients with dementia: baseline results of the DelpHi-trial. Eur. J. Health Econ..

[22] Thyrian JR (2017). Effectiveness and safety of dementia care management in primary care: a randomized clinical trial. JAMA Psychiatry.

[23] Michalowsky B (2019). Cost-effectiveness of a collaborative dementia care management—results of a cluster-randomized controlled trial. Alzheimers Dement.

[24] Byford S, Torgerson DJ, Raftery J (2000). Economic note: cost of illness studies. BMJ.

[25] Bock JO (2015). Calculation of standardised unit costs from a societal perspective for health economic evaluation. Gesundheitswesen.

[26] Ware J, Kosinski M, Keller SD (1996). A 12-item short-form health survey: construction of scales and preliminary tests of reliability and validity. Med. Care.

[27] Brazier JE, Roberts J (2004). The estimation of a preference-based measure of health from the SF-12. Med. Care.

[28] Erzigkeit H (2001). The bayer-activities of daily living scale (B-ADL): results from a validation study in three European countries. Dement. Geriatr. Cogn. Disord..

[29] Michalowsky B (2018). Diagnosing and treating dementia in German primary and specialized care between 2011 and 2015. Int. J. Clin. Pharmacol. Ther.

[30] Michalowsky B (2016). Economic analysis of formal care, informal care, and productivity losses in primary care patients who screened positive for dementia in Germany. J. Alzheimers Dis..

[31] Willan AR, Briggs AH (2006). Statistical analysis of cost-effectiveness data.

[32] Manca A, Hawkins N, Sculpher MJ (2005). Estimating mean QALYs in trial-based cost-effectiveness analysis: the importance of controlling for baseline utility. Health Econ..

[33] Billingham LJ, Abrams KR (2002). Simultaneous analysis of quality of life and survival data. Stat. Methods Med. Res..

[34] Obenchain RL (1999). Resampling and multiplicity in cost-effectiveness inference. J. Biopharm. Stat..

[35] Gomes M (2013). Multiple imputation methods for handling missing data in cost-effectiveness analyses that use data from hierarchical studies: an application to cluster randomized trials. Med. Decis. Making.

