# Supplementary material for: Is the whole larger than the sum of its parts? Impact of missing data imputation in economic evaluation conducted alongside randomized controlled trials
Source: Eur J Health Econ. 2020 Feb 27;21(5):717–28. doi: 10.1007/s10198-020-01166-z (PMC7366573; doi:10.1007/s10198-020-01166-z)
Supplement: Supplementary file 1 — Supplementary file1 (DOCX 80 kb) [file 10198_2020_1166_MOESM1_ESM.docx]

**Supplementary Table 1:** Socio-demographic and clinical characteristics of the patients with complete data in the trial (n=289)

|  | | | Intervention (n=199) | Control (n=90) | *p-value* |
| --- | --- | --- | --- | --- | --- |
| Age | | |  |  |  |
|  | Mean (SD) | | 80.2 (5.5) | 79.6 (4.7) | 0.295^‡^ |
| Sex, n (%) | | |  |  |  |
|  | Female | | 127 (63.8) | 55 (61.1) | 0.902^†^ |
| Living situation, n (%) | | |  |  |  |
|  | | Alone | 106 (53.2) | 41 (45.6) | 0.332^†^ |
| B-ADL | | |  |  |  |
|  | | Mean (SD) | 3.4 (2.4) | 3.1 (2.3) | 0.283^‡^ |
| Number of ICD-10 Diagnoses | | |  |  |  |
|  | | Mean (SD) | 13.8 (8.3) | 14.2 (6.8) | 0.687^‡^ |

B-ADL, Bayer-Activities of Daily Living Scale, range 0-10, lower score indicates better performance; ICD, International Statistical Classification of Diseases and Related Health Problems, SD, standard deviation; ^‡^ t-test; ^†^ Fisher’s exact test.

Supplementary Table 2: Methods for monetary valuation of medical and formal healthcare resources and services

| Cost categories | Services | Units | Unit costs^†^ | Unit cost & source for monetary valuation |
| --- | --- | --- | --- | --- |
| Medical care |  |  |  |  |
| Out-patient physician treatment | GP or specialists | Contact | 20.95€ - 81.56€, depending on specialization | Cost per contact [[1](#_ENREF_1)] |
| In-patient treatment | In-hospital treatment and rehabilitation | Days | 593.04€ and 121.85, respectively | Average per diem cost for in-hospital treatment in Mecklenburg-Western Pomerania & for specialization of rehabilitation [[1](#_ENREF_1)] |
| Medications | Regularly prescribed drugs (Rx-drugs) | Quantity | Market prices, 253.58€^‡^ | Pharmaceutical Index of the Scientific Institute of the AOK [[2](#_ENREF_2)] |
| Medical aids | Aids such as tub-lifts, tub-seats, walking sticks, walkers and others | Quantity | Market prices, 168.92€^‡^ | Market prices |
| Formal care |  |  |  |  |
| Ambulatory care | Home care provided by professionals | Quantity/ Contacts | Market prices, 11.48€^‡^ | Market prices for Mecklenburg Western-Pomerania |

GP, general practitioner; AOK, allgemeine Ortskrankenkasse; ADL, activities of daily living; IADL, instrumental activities of daily living; ^‡^ when drugs, aids or services were unknown or market prices were not available; ^†^ inflation included.

Supplementary Table 3: Healthcare resource utilization, cost and quality-adjusted life of the complete data set (n=289)

|  | Intervention (n=199) | Control (n=90) |  |
| --- | --- | --- | --- |
| Healthcare resource use, mean (SD) |  |  | *p value* |
| In-hospital, days | 4.1 (14.8) | 3.2 (6.6) | 0.562 |
| Medications, number | 7.0 (3.5) | 6.4 (2.9) | 0.076 |
| Ambulatory care, visits | 173.3 (377.5) | 138.3 (351.7) | 0.437 |
| Medical aids, number | 5.5 (2.7) | 5.2 (2.75) | 0.225 |
| Physician, visits | 6.2 (3.8) | 6.3 (5.0) | 0.749 |
|  |  |  |  |
| Costs in Euros, mean (SD) |  |  |  |
| In-hospital | 2,567 (9,182) | 1,953 (4,092) | 0.526 |
| Medications | 2,282 (1,124) | 2,051 (922) | 0.076 |
| Ambulatory care | 1,989 (4,334) | 1,588 (4,038) | 0.437 |
| Medical aids | 939 (469) | 870 (464) | 0.225 |
| Physicians | 163 (100) | 168 (132) | 0.749 |
| Total cost | 7,942 (10,930) | 6,632(6,378) | 0.269 |
|  |  |  |  |
| Quality adjusted life years, mean (SD) | 0.771 (0.13) | 0.761(0.12) | 0.056 |
| Incremental costs, mean (SE) | **1,311 €** (1,185) | |  |
| Incremental QALY, mean (SE) | **+0.010 QALY** (0.02) | |  |
| Incremental cost per QALY | **129,002 €/ QALY** | |  |

SD, standard deviation; SE, standard error; QALY, quality-adjusted life years.

**Supplementary Document 1:** Methods used to handle missing data and used STATA code

To handle missing data at random, we used multiple Imputation by Chained Equations (MICE) [[3-5](#_ENREF_3)]. Mechanism of Multiple Imputations is described in detail by Little & Rubin [[6](#_ENREF_6)]. Using MICE, we specify one imputation model for each variable. For each resource utilization item we used a Poisson regression and for domains of HRQoL an ordered logistic regression (non-aggregated level). Aggregated health utilities and total cost were imputed using a linear regression (aggregated level). We further adjusted each model for age, sex, living situation (alone vs. not alone), comorbidity (number of ICD-10 diagnoses) and functional impairment (bayer activities of daily living). For the imputation of missing domains of HRQoL and utility values, we further adjusted for baseline value. Due to the fact that health resource utilization was assessed retrospective for a period of 12 months, we only imputed missing values at the first follow-up and not for baseline.

An imputation model for the intervention and control group together would only recognize differential means by the study group but not a differential covariance structure. Therefore, MICE were implemented separately by randomization treatment allocation for all missing values of the first and second follow-ups. Missing values of the baseline were imputed without any stratification for study groups [[5](#_ENREF_5)]. 50 additional data sets for each missing variable were generated. Estimates obtained from each imputed data set were combined using Rubin´s Rule [[6](#_ENREF_6)] to generate an overall mean estimate together with its standard error [[7](#_ENREF_7)]. The used STATA code was as follows:

**Imputation of domains of health related quality of life (*Q_i_*):**

mi set flong

mi register imputed ***q***

mi xtset ***cluster_j_***

mi impute chained (ologit) ***q_i_*** = ***var****_baseline_* ***var****_gender_* ***var****_age_* ***var****_living_situation_* ***var****_comorbidity_* ***var****_functional impairment_*, add(50)

egen ***q_i_mean_***= mean(***Q_i_***) by(***var_pat_id_***)

replace ***q_i_***= ***q_i0_mean_*** if ***q_i_***==.

mi unregister ***q***

**Imputation of health resource use items (*RU_i_*):**

mi set flong

mi register imputed ***RU_item_***

mi xtset ***cluster_j_***

mi impute chained (poisson) ***RU_i_***= ***var****_gender_* ***var****_age_* ***var****_living_situation_* ***var****_comorbidity_* ***var****_functional impairment_* if ***d***==[0;1], add(50)

egen ***RU_i_mean_***= mean(***RU_i1_***) if ***d***==[0;1], by(***var_pat_id_***)

replace ***RU_i_***= ***RU_i_mean_*** if ***RU_i_***==. & ***d***==[0;1]

mi unregister ***RU_item_***

**Imputation of health utilities (*Q_i_*):**

mi set flong

mi register imputed ***Q***

mi xtset ***cluster_j_***

mi impute chained (regress) ***Q_i_*** = ***var****_baseline_* ***var****_gender_* ***var****_age_* ***var****_living_situation_* ***var****_comorbidity_* ***var****_functional impairment_*, add(50)

egen ***Q_i_mean_***= mean(***Q_i_***) by(***var_pat_id_***)

replace ***Q_i_***= ***Q_i0_mean_*** if ***Q_i_***==.

mi unregister ***Q***

**Imputation of total cost (*C_i_*):**

mi set flong

mi register imputed ***C***

mi xtset ***cluster_j_***

mi impute chained (regress) ***C_i_***= ***var****_gender_* ***var****_age_* ***var****_living_situation_* ***var****_comorbidity_* ***var****_functional impairment_* if ***d***==[0;1], add(50)

egen ***C_i_mean_***= mean(***C_i1_***) if ***d***==[0;1], by(***var_pat_id_***)

replace ***C_i_***= ***C_i_mean_*** if ***C_i_***==. & ***d***==[0;1]

mi unregister ***C***

$$\boldsymbol{q}_{\boldsymbol{ij}} =Value of the domain of HRQoL of patient \boldsymbol{i} in cluster\boldsymbol{j}$$

| $\boldsymbol{Q}_{\boldsymbol{ij}} =Health utility of patient \boldsymbol{i}in cluster\boldsymbol{j}$ |
| --- |
| $\boldsymbol{RU}_{\boldsymbol{ij}} =Health utilization of patient \boldsymbol{i} in cluster\boldsymbol{j}$  $\boldsymbol{C}_{\boldsymbol{ij}} =Total cost of patient \boldsymbol{i} in cluster\boldsymbol{j}$  $\boldsymbol{q}_{\boldsymbol{ij\_mean}} =Mean estimated value of the domain of HRQoL of patient \boldsymbol{i}in cluster\boldsymbol{j}$ |
| $\boldsymbol{Q}_{\boldsymbol{ij\_mean}} =Mean estimated health utility of patient \boldsymbol{i}in cluster\boldsymbol{j}$ |
| $\boldsymbol{RU}_{\boldsymbol{itj\_mean}} =Mean estimated health utility of patient \boldsymbol{i}in cluster\boldsymbol{j}$  $\boldsymbol{RU}_{\boldsymbol{itj\_mean}} =Mean total cost of patient \boldsymbol{i}in cluster\boldsymbol{j}$ |
| $\boldsymbol{d}_{\boldsymbol{ij}} =Treatment dummy variable \left( 0=control group; 1=intervention \right)$ |

**Supplementary Table 4:** Missing data analysis

|  | **Drop-out/death overall**  **(n=118, 28.9%)** | |
| --- | --- | --- |
|  | **OR (CI)** | **p-value** |
| Study group (Ref. controls) | 0.51 (0.01 7.88) | 0.325 |
| Age | 1.01 (0.96 1.05) | 0.714 |
| Sex (Ref. female) | 1.59 (0.92 2.74) | 0.094 |
| Comorbidity | 0.96 (0.92 1.01) | 0.056 |
| Depression (GDS) | 1.06 (0.92 1.23) | 0.353 |
| Activities in daily living (B-ADL) | **1.26 (1.13 1.41)** | **0.001** |

Multivariate logistic regression analysis with random effects for the general practitioner, univariate odds ratios (OR) for the subsample of n=289 patients.; OR, odds ratios; Ref, reference; CI, confidence interval; MMSE, Mini-Mental State Examination, Range 0-30, higher score indicates better cognitive function; B-ADL, Bayer-Activities of Daily Living Scale, range 0-10, lower score indicates better performance; GDS, Geriatric Depression Scale, sum score 0-15, score ≥ 6 indicates depression; comorbidity, number of ICD-10 diagnoses listed in the general practitioner files.

**Supplementar Table 5:** Distribution of missing values (simulation study based on n=289 patients)

|  | ***BOTH QUESTIONNAIRES*** | | ***SF-6D QUESTIONNAIRE*** | | | | | | | | | ***RESOURCE UTILIZATION QUESTIONNAIRE*** | | | | | | |
| --- | --- | --- | --- | --- | --- | --- | --- | --- | --- | --- | --- | --- | --- | --- | --- | --- | --- | --- |
|  | *Missing Cases (aggregate level)* | | *Missing values per case* | | *% of patients with 1 to 7 items missing* | | | | | | | *Missing values per case* | | *% of patients with 1 to 5 items missing* | | | | |
|  | mean | *range* | *mean^1^ (sd)* | *range^2^* | *1* | *2* | *3* | *4* | *5* | *6* | *7* | *mean^1^ (sd)* | *range^2^* | *1* | *2* | *3* | *4* | *5* |
| **Missing completely at random**  **(MCAR)** | 11.7% | 7% - 17% | 1.09 (0.29) | 1 – 2 | 6.8% | 0.3% | 0% | 0% | 0% | 0% | 0% | 1.05 (0.19) | 1 – 2 | 5.8% | 0.1% | 0% | 0% | 0% |
|  | 21.2% | 16% - 26% | 1.16 (0.39) | 1 – 3 | 11.9% | 1.3% | 0.1% | 0% | 0% | 0% | 0% | 1.11 (0.32) | 1 – 3 | 11.1% | 0.5% | 0.1% | 0% | 0% |
|  | 39.8% | 32% - 47% | 1.39 (0.61) | 1 – 4 | 18.8% | 3.2% | 0.2% | 0.1% | 0% | 0% | 0% | 1.22 (0.47) | 1 – 3 | 20.4% | 2.2% | 0.1% | 0% | 0% |
| **Missing at random**  **(MAR)** | 10.8% | 7% - 14% | 1.14 (0.36) | 1 – 3 | 5.2% | 0.4% | 0.1% | 0% | 0% | 0% | 0% | 1.09 (0.28) | 1 – 2 | 5.6% | 0.3% | 0% | 0% | 0% |
|  | 19.2% | 13% - 23% | 1.33 (0.55) | 1 – 4 | 9.1% | 1.3% | 0.1% | 0.1% | 0% | 0% | 0% | 1.22 (0.46) | 1 – 3 | 9.7% | 0.9% | 0.1% | 0% | 0% |
|  | 38.6% | 33% - 44% | 1.77 (0.82) | 1 – 5 | 17.9% | 5.1% | 0.7% | 0.1% | 0.1% | 0.0% |  | 1.55 (0.57) | 1 – 4 | 17.1% | 3.6% | 0.3% | 0.1% | 0% |
| **Missing not at random**  **(MNAR)** | 5.4% | 3% - 8% | - | - | - | - | - | - | - | - |  | 1.06 (0.22) | 1 – 2 | 5.2% | 0.2% | 0% | 0% | 0% |
|  | 10.8% | 7% - 15% | - | - | - | - | - | - | - | - |  | 1.12 (0.13) | 1 – 4 | 10.2% | 0.5% | 0.1% | 0% | 0% |
|  | 19.8% | 15% - 25% | - | - | - | - | - | - | - | - |  | 1.24 (0.32) | 1 – 5 | 16.5% | 3.2% | 0.1% | 0.1% | 0% |

^1^ average mean number of items missing per incomplete case over 300 reputations; ^2^ range of the number of items missing per incomplete case over 300 reputations

**Supplementary Table 6:** Deviation and range of deviation to true cost-effectiveness acceptability curve

|  |  | **Item Imputation** | | | | | | **Aggregate Imputation** | | | | | |
| --- | --- | --- | --- | --- | --- | --- | --- | --- | --- | --- | --- | --- | --- |
|  |  | Relative bias from true CEAC at WTP of 0€/ QALY | | Relative bias from true CEAC at WTP of 100.000€/ QALY | | Relative bias from true CEAC at WTP of 200.000€/ QALY | | Relative bias from true CEAC at WTP of 0€/ QALY | | Relative bias from true CEAC at WTP of 100.000€/ QALY | | Relative bias from true CEAC at WTP of 200.000€/ QALY | |
|  |  | *mean* | *range^1^* | *mean* | *range^1^* | *mean* | *range^1^* | *mean* | *range^1^* | *mean* | *range^1^* | *mean* | *range^1^* |
| **Missing completely at random**  **(MCAR)** | 10% | 2% | -3% − 6% | 1% | -5% − 7% | 1% | -3% − 6% | 3% | -7% − 17% | 2% | -15% − 18% | 2% | -11% − 13% |
|  | 20% | 2% | -3% − 7% | 1% | -6% − 11% | 1% | -5% − 8% | 3% | -8% − 26% | 3% | -16% − 23% | 3% | -12% − 15% |
|  | 40% | 2% | -5% − 12% | 0% | -11% − 11% | 0% | -8% − 9% | 4% | -10% − 39% | 3% | -24% − 29% | 3% | -16% − 19% |
| **Missing at random**  **(MAR)** | 10% | 2% | -2% − 6% | 1% | -5% − 6% | 1% | -4% − 5% | 4% | -7% − 18% | 2% | -10% − 17% | 2% | -9% − 13% |
|  | 20% | 2% | -3% − 8% | 1% | -6% − 8% | 1% | -5% − 6% | 4% | -7% − 21% | 4% | -11% − 20% | 4% | -7% − 15% |
|  | 40% | 3% | -4% − 13% | 1% | -8% − 12% | 1% | -6% − 9% | 7% | -9% − 38% | 8% | -14% − 29% | -7% | -11% − 21% |
| **Missing not at random**  **(MNAR)** | 10% | 2% | -3% − 6% | 1% | -5% − 6% | 1% | -2% − 5% | 4% | -7% − 23% | 1% | -13% − 16% | 1% | -7% − 9% |
|  | 20% | 2% | -5% − 15% | 1% | -7% − 15% | 1% | -4% − 7% | 4% | -10% − 25% | 2% | -17% − 20% | 2% | -9% − 10% |
|  | 40% | 2% | -7% − 16% | 1% | -12% − 16% | 1% | -5% − 8% | 5% | -10% − 37% | 2% | -19% − 25% | 2% | -10% − 13% |

**1. Bock, J.O., et al., *[Calculation of standardised unit costs from a societal perspective for health economic evaluation].* Gesundheitswesen, 2015. 77(1): p. 53-61.**

**2. WIdO (Wissenschaftliches Institut der AOK) *GKV-Arzneimittelindex*.** [**http://www.wido.de/amtl_atc-code.html**](http://www.wido.de/amtl_atc-code.html)**, 2016.**

**3. van Buuren, S., *Multiple imputation of discrete and continuous data by fully conditional specification.* Stat Methods Med Res, 2007. 16(3): p. 219-42.**

**4. Schafer, J.L. and J.W. Graham, *Missing data: our view of the state of the art.* Psychol Methods, 2002. 7(2): p. 147-77.**

**5. Faria, R., et al., *A guide to handling missing data in cost-effectiveness analysis conducted within randomised controlled trials.* Pharmacoeconomics, 2014. 32(12): p. 1157-70.**

**6. Rubin, D.B. and N. Schenker, *Multiple imputation in health-care databases: an overview and some applications.* Stat Med, 1991. 10(4): p. 585-98.**

**7. Michalowsky, B., et al., *Economic Analysis of Formal Care, Informal Care, and Productivity Losses in Primary Care Patients who Screened Positive for Dementia in Germany.* J Alzheimers Dis, 2016. 50(1): p. 47-59.**
